# Prediction and Analysis of CO_2_ Emission in Chongqing for the Protection of Environment and Public Health

**DOI:** 10.3390/ijerph15030530

**Published:** 2018-03-16

**Authors:** Shuai Yang, Yu Wang, Wengang Ao, Yun Bai, Chuan Li

**Affiliations:** National Research Base of Intelligent Manufacturing Service, Chongqing Technology and Business University, Chongqing 400067, China; yu_wang1994@outlook.com (Y.W.); aowg@163.com (W.A.); yunbai@foxmail.com (Y.B.); chuanli@21cn.com (C.L.)

**Keywords:** public health, CO_2_ emission, environmental protection, energy consumption, SVR prediction

## Abstract

Based on the consumption of fossil energy, the CO_2_ emissions of Chongqing are calculated and analyzed from 1997 to 2015 in this paper. Based on the calculation results, the consumption of fossil fuels and the corresponding CO_2_ emissions of Chongqing in 2020 are predicted, and the supporting data and corresponding policies are provided for the government of Chongqing to reach its goal as the economic unit of low-carbon emission in the ‘13th Five-Year Plan’. The results of the analysis show that there is a rapid decreasing trend of CO_2_ emissions in Chongqing during the ‘12th Five-Year Plan’, which are caused by the adjustment policy of the energy structure in Chongqing. Therefore, the analysis and prediction are primarily based on the adjustment of Chongqing’s coal energy consumption in this paper. At the initial stage, support vector regression (SVR) method is applied to predict the other fossil energy consumption and the corresponding CO_2_ emissions of Chongqing in 2020. Then, with the energy intensity of 2015 and the official target of CO_2_ intensity in 2020, the total fossil energy consumption and CO_2_ emissions of Chongqing in 2020 are predicted respectively. By the above results of calculation, the coal consumption and its corresponding CO_2_ emissions of Chongqing in 2020 are determined. To achieve the goal of CO_2_ emissions of Chongqing in 2020, the coal consumption level and energy intensity of Chongqing are calculated, and the adjustment strategies for energy consumption structure in Chongqing are proposed.

## 1. Introduction

From the evaluation report of Intergovernmental Panel on Climate Change (IPCC), the emission of green gases is confirmed as the primary reason for global warming. With the climate change caused by global warming, the development of human society is threatened by natural disasters such as sea level rise and extreme weather [1,2]. According to ‘Global Climate Risk Index 2018’, more than 524,000 people died worldwide as a direct result of more than 11,000 extreme weather events, and the loss in Purchasing Power Parities (PPP) has reached US$3.16 trillion at the time span of 1997 to 2016 [2,3,4,5]. Without any doubt, global warming has become one of the top environmental hazard all over the world. It is well known, global warming is mainly caused by the excessive emissions of greenhouse gases, which includes carbon dioxide (CO_2_), methane, nitrous oxide, and fluorine gas [6]. With its dominant role in the growth of green gases emissions, CO_2_ is generated by fossil fuels and other related industrial processes [7]. Based on previous studies, CO_2_ emissions will also cause the generation of other pollutants like sulfur dioxide and nitrogen dioxide, which are extremely hazardous to public health [6,8]. Therefore, for the protection of the environment and public health, the CO_2_ emissions caused by fossil-fuel consumption need to be controlled.

China is the largest growth market for energy in this world, which accounts for 23% of global energy consumption, and 27% percent of global energy consumption growth [9]. Therefore, for China, the control of the greenhouse gases emissions will be a significant factor to stop global warming. As an important member states of the ‘Kyoto Protocol’, efforts have been made by China in the task of low-carbon emission reduction and building an environment-friendly society. During the ‘12th Five-Year Plan’ (FYP12:2011–2015), the first batch of low-carbon pilot cities were promoted by China to achieve the goal of environment-friendly society. By classifying a city as an economic unit, the reasonable reduction policy of CO_2_ emission has been proven to be an effective way to stimulate the growth of the low-carbon economy. In ‘19th Communist Party of China National Congress’, the coordinated development of green cycle and low carbon is proposed again, which shows the determination of Chines government for the energy conservation and emission reduction.

As the largest heavy industrial city in southwest China, the energy consumption structure of Chongqing is mainly based on fossil energy and electricity. In 2010, Chongqing is defined as the first low-carbon pilot city by Chinese National Development and Reform Commission. In response to the national policy of environment-friendly society, a work plan for greenhouse gas emission control during the ‘13th five-year plan’ (FYP13:2016–2020) period is issued by the Chongqing government. In this plan the CO_2_ emissions per unit of GDP in Chongqing will be dropped by more than 19.5% by 2020 compared to 2015 [10]. Hence, to achieve the goal of low CO_2_ emissions, research about the status and law of CO_2_ emissions in Chongqing need to be carried out, which will play a significant role in strategy of development in China.

In the last two decades, the progress achieved by researchers regarding to the research of CO_2_ emission is impressive [7,11,12,13,14,15,16,17,18,19,20,21,22]. Davis et al. presented a global consumption-based CO_2_ emissions inventory and calculations of associated consumption-based energy and carbon intensity [7]. Heil estimated the historic relationship between carbon emissions and GDP in his study [11]. By taking India as an example, Tiwari indicated the relationship between energy consumption, CO_2_ emissions and economic growth in this country [12]. From the energy point of view, the CO_2_ emission factors in Guangdong Province were analyzed by Wang et al. [13]. There is any little difference in the methodology of prediction on carbon emissions throughout the world. Among them, EKC curve [14,15], STIRPAT model [16], LMDI decomposition [17,18], genetic Algorithm [19], support vector machine stands for the mainstream research method [20]. Ikaga et al. estimated buildings-related CO_2_ emissions in Japan up to 2050 [21]. Panareda et al. analyzed global atmospheric CO_2_ [22]. In this study, a comparison of the ability to predict changes in simulated CO_2_ changes at different time and space scales with observations was proposed. Despite the progress of the prediction on CO_2_ emission, the relative research about analysis and decision-making based on the prediction results is rare.

In this paper, the prediction of CO_2_ emissions based on energy consumption is proposed, as well as the adjustment strategies for energy consumption structure in Chongqing. It is organized as follows: in Section 2, the relevant data are collected and analyzed, and the corresponding mathematical method is introduced. In Section 3, energy consumption of Chongqing from 2016 to 2020 is predicted by SVR, and the corresponding CO_2_ emissions are calculated. To achieve the goal of measuring CO_2_ emissions of Chongqing in 2020, the coal consumption level and energy intensity of Chongqing are calculated in Section 4, and the adjustment strategies for energy consumption structure in Chongqing are proposed. The conclusions are drawn in Section 5.

## 2. Data Analysis and Mathematical Method for Prediction 

### 2.1. Data Analysis

#### 2.1.1. Energy Intensity and CO_2_ Intensity

Due to a lack of official statistics, energy intensity is used to calculate the total energy consumption of Chongqing under different scenario assumptions, and CO_2_ intensity is used to calculate the CO_2_ emission target of the Chongqing government. Energy intensity is the ratio between total primary energy consumption/final energy use and gross domestic product. It reflects the economic benefits of energy consumption. The CO_2_ intensity in this paper is defined as the emissions of CO_2_ per unit of GDP [23,24]. The function of energy intensity is written as follows:

(1) $$I_{e}=\frac{E_{t}}{G_{t}}$$

where $I_{e}$ represents the energy intensity, $E_{t}$ stands for the energy consumption emissions, $G_{t}$ is the value of GDP.

The function of CO_2_ intensity can be described as follows:

(2) $$I_{c}=\frac{C_{t}}{G_{t}}$$

where $I_{c}$ is the energy intensity, $C_{t}$ is the CO_2_ emissions.

#### 2.1.2. Energy Consumption in Chongqing

In this paper, all the data is cited from the National Bureau of Statistics of the People’s Republic of China and Chongqing Statistics [25,26]. As a typical model of conventional energy consumption, the main energy consumption in Chongqing is fossil fuels, such as coal, oil and natural gas. Due to its geographical condition, the dominating clean power in Chongqing is hydroelectric power. Coal plays a dominant role in the energy consumption of Chongqing, which is accounting for 57.68% of total energy consumption. The proportions of hydroelectric power, natural gas and oil are 13.32%, 14.43% and 14.57% respectively. Among them, there are up to 86% energy consumption in Chongqing is generated by fossil fuels. To the best of our knowledge, most CO_2_ emissions are produced by fossil fuels, which will be discussed in this paper. Thus, the energy consumption in the following paper refers to fossil energy consumption.

According to National Bureau of Statistics of the People’s Republic of China, the fossil energy consumption is divided into eight categories which are coal, coke, gasoline, diesel oil, crude oil, kerosene, fuel oil, and natural gas, respectively. Since Chongqing was appointed as a municipality city by central government of China in 1997, the economic policy was adjusted at that time point, this paper only selects the related data from 1997 to 2015.

**Figure 1:**
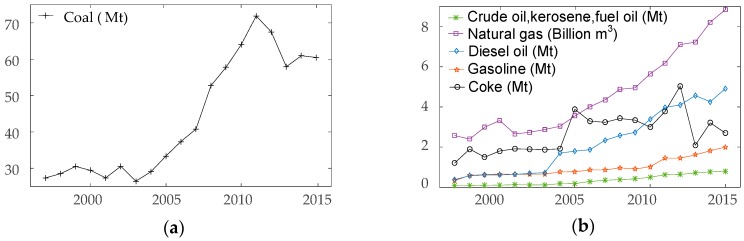
The fossil energy consumption of Chongqing from 1997 to 2015. (**a**) Coal consumption; (**b**) The others energy consumption. In this paper, the unit of natural gas is ‘Billion m^3^’, the other energy is ‘Mt’.

Due to the low consumption of crude oil, kerosene and fuel oil in Chongqing, these three types of fossil fuels are combined as one category in the future discussion. As shown in Figure 1, the coal consumption trend of Chongqing can be divided into three different stages. The first stage is the period from 1997 to 2003, when the coal consumption is stable; Chongqing is a typical industrial city with coal-based energy consumption [27]. During this time span, as a new municipality city, the economic development of Chongqing was at an initial phase. The second stage is the range from 2004 to 2011, the coal consumption of Chongqing shows a steady increasing trend due to the economic developments. Final stage is time span from 2012 to 2015, the coal consumption of Chongqing shows a rapid decreasing trend. Chongqing was appointed as a low-carbon pilot city in 2010, the energy-saving and emission-reduction policies of Chongqing government caused a series significant impacts on coal consumption in Chongqing. The consumption of gasoline, diesel oil, natural gas and kerosene, crude oil, fuel oil have been increasing since 1997. Among them, the largest growth rate of energy consumption is diesel oil, which is followed by gasoline.

Table 1 presents the energy intensity of Chongqing and the ratio between each energy source and total energy consumption in Chongqing from 1997 to 2015. Based on Table 1, the trend of energy intensity declines with the time in Chongqing. From 1997 to 2015, the average coal consumption is responsible for 70% of the total fossil energy consumption. Although the total quantity of coal consumption has been decreasing year by year, it is still the primary energy consumption in Chongqing. The ratios of natural gas and diesel oil consumption have been increasing after 2011.

#### 2.1.3. Calculation and Analysis of CO_2_ Emission 

Due to the lack of official data for CO_2_ emissions, relevant methods need to be applied for the calculation. In this paper, coal, coke, gasoline, diesel oil, crude oil, kerosene, fuel oil, and natural gas are chosen to calculate the CO_2_ emission of Chongqing. The basic calculation formula is given by 2006 IPCC Guidelines for National Greenhouse Gas Inventories, which can be presented as follows [28]:

(3) $$C=\sum_{i=1}^{n}C_{i}=\sum_{i=1}^{n}E_{i}\times F_{i},(n=8)$$

where C represents the emission value of CO_2_, n represents the number of varieties of fossil energy. $E_{i}$ is the *i*th type of energy consumption, and $F_{i}$ stands for the carbon emissions coefficient of the *i*th type energy. According to IPCC Guidelines for National Greenhouse Gas Inventories, the carbon emissions coefficient in Equation (3) can be represented as follows:

(4) $$F_{i}=L_{i}\times T_{i}\times R_{i}\times V_{i}$$

where $L_{i}$ in this equation is the average lower calorific value [29]. $T_{i}$ is the carbon content per unit calorific value, $R_{i}$ represents the rate of carbon oxidation [30]. $V_{i}$ represents the carbon conversion coefficient, which is 44/12 [31]. The following is the CO_2_ emission coefficient used in this paper: 1.9003 (coal), 2.8604 (coke), 3.0202 (crude oil), 2.9251 (gasoline), 3.0179 (kerosene), 3.0959 (diesel oil), 3.1705 (fuel oil), 2.1622 (natural gas).

**Figure 2:**
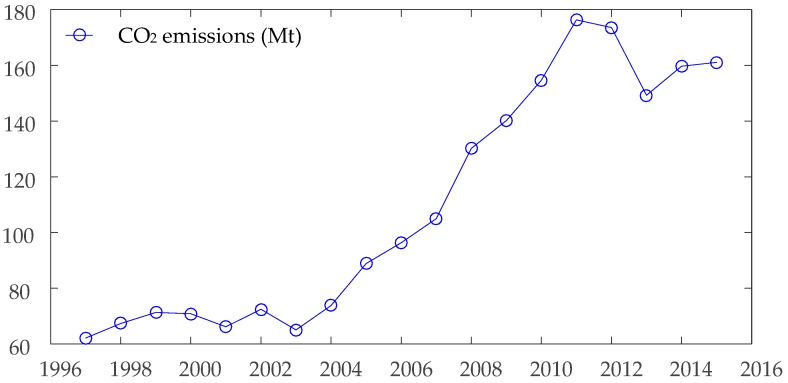
CO_2_ emissions of Chongqing from 1997 to 2015.

According to Equations (3) and (4), the results of CO_2_ emissions in Chongqing from 1997 to 2015 are shown in Figure 2. From this figure, the trend of the CO_2_ emission of Chongqing can be divided into three different stages. The first stage is the range from 1997 to 2003, the CO_2_ emissions of Chongqing are stable, there is no obvious fluctuation during this time span.

**Figure 3:**
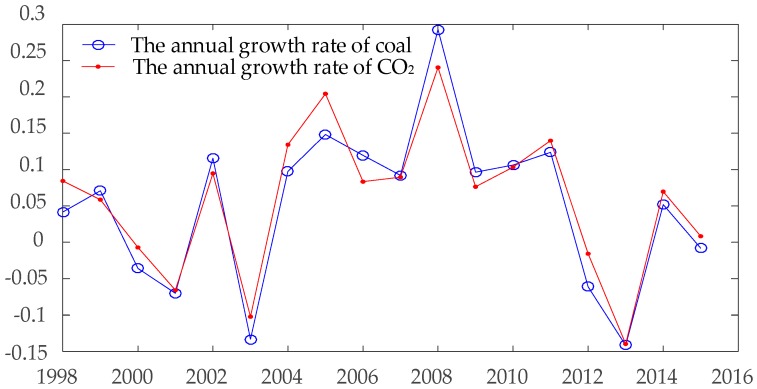
The growth rate of coal and CO_2_ emissions in Chongqing from 1998 to 2015.

The second stage is time span from 2004 to 2010, the CO_2_ emissions of Chongqing show a steady and linear increasing trend through this time range. The finally stage is the range from 2011 to 2015. In the contrary to the former stage, the CO_2_ emissions of Chongqing show a rapid decreasing trend in this time span. The trend of CO_2_ emissions in Chongqing is similar with the coal consumption in the previous analysis. Figure 3 shows the annual growth rate of CO_2_ emissions and coal consumption in Chongqing. The reason for such trend in Chongqing’s CO_2_ emissions can be explained by the change of coal consumption. The formation reasons for the decreasing of coal from 2011 to 2015 in Chongqing are analyzed in a later section of this paper.

**Figure 4:**
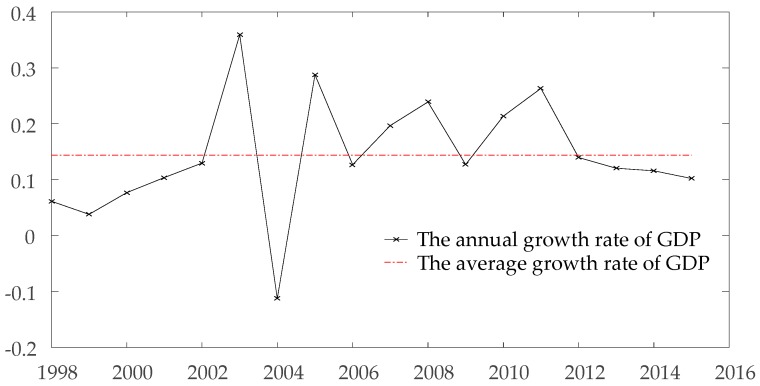
The growth rate of GDP in Chongqing from 1998 to 2015.

As mentioned above, government policy plays a dominant role in the CO_2_ emissions. The energy-saving and emission-reduction policies during ‘12th Five-Year Plan’ of Chongqing government caused a series significant impacts on coal consumption in Chongqing, which led to a sudden reduction in CO_2_ emissions. According to the official report of Chongqing government, a total amount of 16.29 million tons of low-level production capacity was eliminated. Among them, 3 small-size coal mines were permanently closed, 69 coal mines were closed for the optimization of structure, 261 coal mines were upgraded, and 95 coal mines were restructured into 17 model enterprises [32,33]. Without any effect to economic development in Chongqing, CO_2_ emissions of Chongqing is rapidly decreased by these low-carbon policies, which can be observed in Figure 3. Figure 4 shows the growth rate of GDP in Chongqing from 1997 to 2015. From 2012 to 2013, the CO_2_ emissions of Chongqing shows a sudden inclination. In the contrary, the GDP growth rate of Chongqing is still at a health level compare to former several years, which are 13.97% and 12.04% respectively.

### 2.2. Description of Input and Output

Time series prediction is applied to this paper. Supposing that $x_{i}^{*}$ and $y_{i}^{*}$ are the input data and output data respectively. As is known to us, there is a positive correlation between energy consumption and socio-economic development [34]. When the society reaches a certain level, without any major changes (such as technology or policies), the level of energy consumption and social development generally correspond to each other. For example, energy consumption in our present level of social development will not suddenly drop to the level of 10 years ago. As a result, the energy consumption of the current moment is more relevant to the data of neighboring years, and this correlation decreases as the distance of time increases.

Therefore, a sliding time window over time is created. To keep the width of the sliding time window constant, a new sample is added from the right side of the time window and the old sample is shifted out from the left side during modeling. The input data and output data can be rewritten as: $Q=\{(x_{i}^{*},y_{i}^{*})|x_{i}^{*}\in (x_{i},x_{i+1},\ldots ,x_{i+k-1}),y_{i}^{*}\in x_{i+k},i=1,2,\ldots ,19-k\}$, where *k* represents the width of the sliding time window. Autocorrelation analysis is used for the determination of *k* [35,36]. The function can be presented as:

(5) $$m_{k}=\frac{\sum_{i=1}^{n-k}\left(x_{i}-\bar{x})(x_{i+k}-\bar{x}\right)}{\sum_{i=1}^{n}{\left(x_{i}-\bar{x}\right)}^{2}}$$

where $m_{k}$ stands for the coefficient of autocorrelation when the width of sliding time window is k. $\bar{x}$ is the mean of the time series. n represents the number of data.

### 2.3. Support Vector Regression Method for Time Series Prediction

Based on the data analysis above, the trend of CO_2_ emissions in Chongqing is nonlinear, which can be affected by many factors. Among them, the government policy usually plays a significant role [37]. Therefore, by the previous data of CO_2_ emissions in Chongqing, the prediction results can be inaccurate. However, by the prediction of non-fossil energy consumption in Chongqing, the CO_2_ emissions of Chongqing can be predicted indirectly.

Due to the small samples with the characteristics of non-linear, Support Vector Machine method (SVM) is applied to analyze the selected data. SVM is one of machine learning methods based on statistic theory based on statistic theory, which is proposed by Cortes and Vapnik in 1995 [38]. It provides excellent results on solving problem related to small samples, nonlinear and high-dimensional pattern recognition [39]. SVM for regression is also called SVR. By using the thought of support vector and the method of Lagrange multiplier, the data can be analyzed by support vector regression. The basic function for SVR is given by [38,39,40,41,42]:

(6) f(x)=ω⋅∮(x)+b

where f(x) is a nonlinear mapping function, ω and b are the vector parameters need to be determined. With data set $\left\{(x_{1},y_{1}),(x_{2},y_{2}),\ldots ,(x_{n},y_{n})\right\}$ ($y_{i}\in R^{n}$, $x_{i}\in R^{n}$), $x_{i}$ is an input, $y_{i}$ is a output target and $R^{n}$ represents n-dimensional Euclidean space. Function f(x) can be trained to approximate the actual value y. The objective function of support vector regression can be presented as follows:

(7) $$\underset{\omega ,b}{\min}\frac{1}{2}{\left\|\omega \right\|}^{2}+C\sum_{i=1}^{n}\int (f(x_{i})-y_{i})$$

In Equation (6), $\int (f(x_{i})-y_{i})$ is the insensitive loss function, C is the penalty factor, n represents the number of data. The insensitive loss function of SVR is shown as follows:

(8) $$\int =\{\begin{array}{cc} |f(x_{i})-y_{i}|-\varepsilon , & |f(x_{i})-y_{i}|\geq \varepsilon \\ 0, & \mathrm{otherwise} \end{array}$$

where ε stands for insensitive loss parameter. In SVR method, largest ε-deviation can be placed between the predicted value f(x) and the actual value y. When the absolute value of the difference between f(x) and y is bigger than ε, the calculation of the cost value will start. The margin with 2ε width will be constructed under the center of f(x). The point right with the boundary is the determining model of ‘support vector’. If the training sample stays in the margin, the results will can be assumed as correct.

Due to the flexible boundary, the slack variable $\xi_{i}$ and $\xi_{i}^{*}$ are introduced, thus the equation can be rewritten as:

(9) $$\begin{array}{c} \underset{\omega ,b,\xi_{i},\xi_{i}^{*}}{\min}\frac{1}{2}{\left\|\omega \right\|}^{2}+C\sum_{i=1}^{n}\left(\xi_{i}+\xi_{i}^{*}\right)\\ \mathrm{subject}\;\mathrm{to}\;\left\{ \begin{array}{l} y_{i}-f(x_{i})\leq \varepsilon +\xi_{i}\\ f(x_{i})-y_{i}\leq \varepsilon +\xi_{i}^{*}\\ \xi_{i}\geq 0,\;\xi_{i}^{*}\geq 0,\;i=1,2,\ldots ,n \end{array}\right. \end{array}$$

where $\xi_{i}$ and $\xi_{i}^{*}$ represent the upper error limit and the lower error limit, respectively. By Introducing the Lagrange multipliers α≥0, δ≥0, the Lagrange function is:

(10) $$\begin{array}{c} \mathrm{Lg}\left(\omega ,b,\xi ,\alpha ,\delta \right)=\frac{1}{2}{\left\|\omega \right\|}^{2}+C\sum_{i=1}^{n}\left(\xi_{i}+\xi_{i}^{*}\right)-\sum_{i=1}^{n}\delta_{i}\xi_{i}-\sum_{i=1}^{n}\delta_{i}^{*}\xi_{i}^{*}\\ +\sum_{i=1}^{n}\alpha_{i}{(y}_{i}-f(x_{i})-\varepsilon -\xi_{i})+\sum_{i=1}^{n}\alpha_{i}^{*}(f(x_{i})-y_{i}-\varepsilon -\xi_{i}) \end{array}$$

The dual function of Equation (8) can be written as:

(11) $$\begin{array}{c} \underset{\alpha ,\alpha^{*}}{\max}\sum_{i=1}^{n}y_{i}(\alpha_{i}^{*}-\alpha_{i})-\varepsilon (\alpha_{i}^{*}+\alpha_{i})-\frac{1}{2}\sum_{i-1}^{n}\sum_{j=1}^{n}(\alpha_{i}^{*}-\alpha_{i})(\alpha_{j}^{*}-\alpha_{j})K(x_{i},x_{j})\\ \mathrm{subject}\;\mathrm{to}\;\left\{ \begin{array}{l} \sum_{i=1}^{n}(\alpha_{i}^{*}-\alpha_{i})=0\\ 0\leq \alpha_{i},\alpha_{i}^{*}\leq C,i=1,2,\ldots ,n \end{array}\right. \end{array}$$

In Equation (11), function $K(x_{i},x_{j})$ is the kernel function. The function of kernel can transfer the non-linear problem of low-dimensional space into programming linear problem of high-dimensional space. The approximate function is:

(12) $$f(x)=\sum_{i=1}^{n}(\alpha_{i}-\alpha_{i}^{*})K(x_{i},x)+b$$

The chosen of kernel function and parameters will cause obvious difference on the simulation results of SVR [42]. At present, there is no standard method about the choice of kernel function. The most widely used kernel function is Gaussian Radial Basis Function (RBF) [43], which is employed in this paper. The formula can be described as: $K(x_{i},x_{j})=\exp(-g{\left\|x_{i}-x_{j}\right\|}^{2}),g>0.$ where g stands for the RBF function parameter. In this paper, penalty factor C and RBF function parameter g are the parameters need to be determined.

## 3. Simulation and Prediction

As mentioned before, from 2011 to 2015, the CO_2_ emissions of Chongqing and the coal consumption both show a rapid decreasing trend in this time span due to the adjustment of the government policy. From the best of our knowledge, the government policy will not be an eternal way to solve situation like this. Especially for Chongqing, which is the economic unit with the fastest economic growth rate in China. With the developing trend of mega city, the energy consumption of Chongqing will still show a steady increasing trend in the near future.

By using SVR method, the prediction results are based on previous experience, which means under a data set with short fluctuation, the precision of prediction will be deviating. However, from the data in Figure 1b, gasoline, diesel oil, crude oil, kerosene, fuel oil and natural gas have been showing steady ascending trends year by year. Two reasons can be concluded to explain this phenomenon. First, all these six types of energy are related to the basic daily life of citizens in Chongqing. With the increasing of population in Chongqing, these energy consumptions will ascend inevitably. Moreover, from the previous records, policy regarding to these energies did not show obvious effect on their growth rate of consumption.

Therefore, SVR method is applied to predict the consumptions of gasoline, diesel, natural gas, crude oil, kerosene and fuel oil in this paper. By assuming the structure and level of energy consumption are stable in Chongqing, the total energy consumption of Chongqing in 2020 can be obtained based on the energy intensity. With these two calculation results, the both consumptions of coal and coke of Chongqing in 2020 will be predicted indirectly.

### 3.1. Determination of the Inputs and Outputs

In this paper, time-series: gasoline, diesel, natural gas, crude oil, kerosene and fuel oil (in Figure 1b) are selected for the prediction of SVR. The data from 1997 to 2008 are used as the training samples to train the model. The data from 2009 to 2015 are selected to validate the accuracy of the model. Through the process of normalization, the original data is linearly mapped to the interval of (−1, 1) to reduce the amount of computation.

According to Equation (5), when the value of ‘Autocorrelation Function’ (ACF) reaches at 0.5, it is chosen as the width of the sliding time window [44]. Based on the calculation results, 2 is employed as the width to predict the energy consumption of gasoline, and 3 is the width used for prediction of the rest three time series.

### 3.2. Selection of Parameters Used in SVR

The method of grid-search is used to obtain the parameter combination *g* and *C*. The parameters with the highest cross-validation accuracy can be determined. The mean absolute percentage error (MAPE) is used to verify the parameters selected by grid-search and cross-validation. The obtained result of MAPE shows the accuracy of the final results. When the value of MAPE is minimum, the corresponding combination of parameters are selected as the best parameters for the model.

Table 2 shows the RBF function parameters and penalty factors used in this paper. The smallest MAPE in the performance of SVR are 7.16%, 5.70%, 3.76%, 3.99% respectively. When MAPE < 10%, the prediction performance shows high accuracy [45].

### 3.3. Evaluation of Model Accuracy

**Figure 5:**
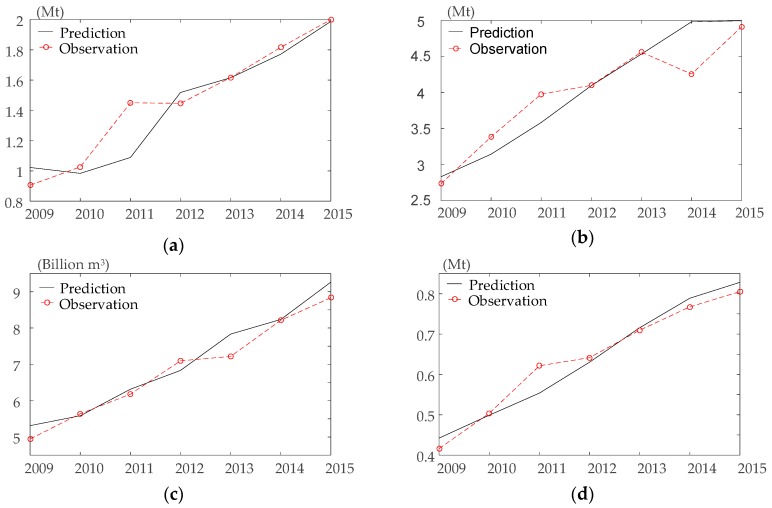
The comparison of observation and the prediction results of SVR. (**a**) Gasoline; (**b**) Diesel oil; (**c**) Natural gas; (**d**) Crude oil, kerosene, fuel oil.

The parameters of Table 2 are applied into the model for validation, the observation and prediction values are compared in Figure 5. The accuracy of the model is validated in this part. The model can be used to predict the data from 2016 to 2020 when it passes the test. In this paper, posteriori error estimation is introduced to evaluate the accuracy of SVR method [46]. The main steps are described as follows:

Step 1: The residual sequences between observation and prediction value are calculated as e. Where $x_{o}$ and $x_{p}$ stands for the value of observation and prediction, respectively:

(13) $$e=x_{o}-x_{p}$$

Step 2: The standard deviation of residual and observation sequences are calculated as $S_{o}$ and $S_{e}$:

(14) $$S_{o}=\sqrt{\frac{\sum_{i=1}^{n}{\left[x_{o}(i)-\bar{x_{o}}\right]}^{2}}{n-1}}$$

(15) $$S_{e}=\sqrt{\frac{\sum_{i=1}^{n}{\left[e(i)-\bar{e}\right]}^{2}}{n-1}}$$

Step 3: According to Equations (15) and (16), the posterior error ratio D and error probability P can be obtained [46,47]:

(16) $$D=S_{e}{/S}_{o}$$

(17) $$P=p\left\{\;|e(i)-\bar{e}\;|<0.6745S_{o}\right\}$$

Through calculation, the accuracy of the model is shown in Table 3. The rating grade of model accuracy is shown in Table 4 [46]. Based on the above analysis, the model can be applied to predict the data from 2016 to 2020.

### 3.4. Prediction Result with Analysis

In this part, the model which has been validated in previously part is employed to predict the consumption of gasoline, diesel oil, natural gas, crude oil, kerosene and fuel oil in Chongqing from 2016 to 2020. The prediction results can be seen from Table 5. According to the results, these types of energy consumption in Chongqing will keep a steady growth trend in the next five years. Through Equations (3) and (4), the CO_2_ emissions of Chongqing in 2020 can be determined by the energy consumptions of gasoline, diesel, natural gas and crude oil, kerosene and fuel oil in 2020. The calculation results are shown in Table 6.

According to Equation (1), the total fossil energy consumption of Chongqing in 2020 can be predicted by energy intensity with the given GDP target of Chongqing. In the official reports of Chongqing government, the GDP of Chongqing should reach to 2500 billion yuan in 2020. By assuming the current level of energy intensity will be maintained, the total energy consumption of Chongqing in 2020 can be obtained by the energy intensity of Chongqing in 2015. With the reduction of the determined CO_2_ emissions from gasoline, diesel, natural gas and crude oil, kerosene and fuel oil, the consumption of coal and coke under the same energy intensity can be obtained as well as the corresponding CO_2_ emissions of Chongqing. The results are shown in Table 7. With the energy intensity of 2015, the CO_2_ emissions caused by coal and coke will be 205.08 (Mt) tons in 2020. Comparing to 2015, the CO_2_ emissions of Chongqing will increase 82.44 (Mt).

## 4. Analysis

In the previous analysis, the energy consumption (except for coal and coke) of Chongqing in 2020 are predicted, as well as the corresponding CO_2_ emissions. Therefore, the CO_2_ emissions within government target of coal and coke can be calculated. This section focuses on the analysis of the CO_2_ emissions and consumption of coal and coke under different energy intensity scenarios.

Based on the official report of Chongqing government, the CO_2_ emissions of per unit GDP in Chongqing will drop by more than 19.5% compare to 2015 in 2020. According to this target, the target value of Chongqing’s CO_2_ emissions in 2020 can be determined. By reduction of predicted CO_2_ emissions of gasoline, diesel, natural gas and crude oil, kerosene and fuel oil in the same time, the CO_2_ emissions of coal and coke from Chongqing in 2020 can be obtained under the same energy intensity. The results are shown in Table 8. To achieve the proposed goal of CO_2_ emissions in Chongqing, the CO_2_ emissions caused by coal and coke need to be under 151.48 (Mt) by 2020.

According to the results given in Table 7, when the energy intensity of Chongqing in 2020 is maintained at the level of 2015, the CO_2_ emissions of coal and coke in Chongqing will reach 205.76 (Mt). This result is 54.28 (Mt) higher than the proposed CO_2_ emissions of Chongqing government given in Table 8. Therefore, to reduce the CO_2_ emissions in Chongqing and reach the official target of CO_2_ emissions in 2020, the energy intensity need to be reduced. In order to reach the official GDP target, the reduction of total energy consumption is the only method to decrease the energy intensity.

Liang et al. proposed an analysis on the consumption of Chinese energy industry [48]. According to this research, industry with the highest energy consumption is manufacturing industry, which is also the largest coal consumption industry. Although various industries are trying to reduce their coal consumption, coal will still be one of the most important energy source of Chongqing in the near future. The consumption of gasoline increases in industries like transportation, warehousing and telecommunications instead of manufacturing industry. Like gasoline, the trend of diesel oil consumption also deviates to transportation and other industries. The primary consumption of natural gas is for daily lives of citizens in our country, the ratio of application for industry is small [47].

Based on the previous analysis, the applications of different energy sources are demonstrated. With the GDP growth and the development of tertiary industry in Chongqing, the energy consumption regarding to daily lives of citizens will inevitably increase, which will eventually lead to the rising energy consumption of gasoline, diesel, natural gas, crude oil, kerosene and fuel oil. Therefore, to reduce the total amount of energy consumption in Chongqing, the consumption of coal and coke should be the primary target, which has been verified by the adjustment results of energy in Chongqing during ’12th Five-Year plan’.

The coal consumption and CO_2_ emissions of coal in Chongqing under the different average annual declinations of energy intensity can be calculated through the above method. Table 9 shows the calculation results. From Table 9, when the annual declination of energy intensity is 4%, CO_2_ emissions of coal in Chongqing will reach to the limitation of the official target of Chongqing government, and the corresponding coal consumption is 79.49 (Mt). Therefore, to achieve the reduction plan of CO_2_ emissions in 2020, the coal consumption of Chongqing should be limited in 79.49 (Mt).

## 5. Conclusions

Based on the prediction and analysis of fossil energy consumption and CO_2_ emissions in Chongqing, this paper reveals its inherent variation of CO_2_ emissions and focuses on the impact of coal consumption on them. To achieve low-carbon development of Chongqing, a low-carbon energy system needs to be built for the promotion of energy structure optimization. Due to the dominant ratio of coal consumption of the fossil energy in Chongqing, there are several effective ways to optimize the energy structure in Chongqing by the reduction of the coal consumption, such as increasing the consumption of natural gas, development of wind power, development of solar energy and other renewable energy sources. According to the analysis results, the specific suggestions for Chongqing to achieve its reduction targets of CO_2_ emission are proposed as follows:

1. The price leverage can be applied to control the total amount of coal consumption by government. Meanwhile, the supplement of non-fossil fuels should be adequate, and the consumption of high-quality energy need to be encouraged, such as renewable energy, natural gas and electricity. Due to the geographical feature, the storage of natural gas in Chongqing is abundant. In the contrary, the supply systems of natural gas in Chongqing is under a lagging development relatively. Therefore, the fundamental constructions for these renewable energies are necessary.
2. By enhancing the management of the energy consumption of high-energy consumption industry, the CO_2_ emissions need to be limited during industrial production process. Especially for the top five carbon-intensive and high-consumption of coal enterprise, which are thermal power, iron steel, non-ferrous metals, chemical industry and construction materials. The efficiency and cleaning-level of energy consumption should be improved to control the consumption of coal.
3. The applications of high-quality and clean coal need to be promoted by government. By the cost reduction of clean coal, the promotion of application for clean coal is a primary strategy to achieve the goal of low CO_2_ emissions. Meanwhile, through eliminating production capacity with low-efficiency, the problem of excess production capacity will be receded, which will lead to the relief of energy pressure.

As the first low-carbon pilot city, the contributions on the reduction of CO_2_ emissions that was made by Chongqing will play a significant role in strategy of development in China. Tremendous contributions have been made by China to develop the green economy and build an environment-friendly society. In the future, to promote the work of emission reduction, the experience that gained at one unit should be popularized in a whole area.

## Figures and Tables

**Table 1:** Energy intensity in Chongqing from 1997 to 2015 and the proportion of each energy source to total energy consumption.

Year	Energy Intensity	Coal (%)	Coke (%)	Gasoline (%)	Diesel Oil (%)	Kerosene, Crude Oil, Fuel Oil (%)	Natural Gas (%)
1997	1.67	77.26	4.63	1.91	2.20	0.45	13.54
1998	1.70	74.75	6.78	3.22	3.08	0.45	11.72
1999	1.76	74.66	4.98	3.17	3.05	0.50	13.63
2000	1.63	71.93	6.01	3.31	3.06	0.55	15.14
2001	1.37	72.39	6.87	3.50	3.47	0.68	13.09
2002	1.32	74.11	6.26	3.27	3.41	0.60	12.35
2003	0.88	70.64	6.82	3.62	3.98	0.65	14.29
2004	1.13	68.05	6.10	3.69	8.10	0.83	13.24
2005	1.05	65.53	10.37	3.14	7.22	0.75	12.99
2006	1.01	67.35	8.07	3.20	6.86	1.07	13.45
2007	0.93	67.35	7.26	2.94	7.89	1.18	13.38
2008	0.92	70.78	6.25	2.67	7.06	1.06	12.18
2009	0.87	72.39	5.69	2.34	6.99	1.07	11.53
2010	0.80	72.20	4.60	2.39	7.79	1.17	11.86
2011	0.72	71.24	5.09	2.96	8.04	1.26	11.40
2012	0.63	67.35	6.84	2.97	8.34	1.31	13.19
2013	0.49	65.61	3.22	3.77	10.53	1.65	15.22
2014	0.47	64.43	4.61	3.95	9.18	1.66	16.17
2015	0.44	62.74	3.81	4.27	10.40	1.71	17.07

**Table 2:** The RBF function parameters and penalty factors used in SVR.

Energy Type	*g*	*C*	MAPE (%)
Gasoline	1505.70	4553.73	7.16
Diesel oil	3723.09	955.36	5.70
Natural gas	5594.36	1102.49	3.76
Crude oil, kerosene, fuel oil	2543.05	119.60	3.99

**Table 3:** The calculation result of model accuracy.

Time Series Type	*p*	*D*
Gasoline	0.86	0.39
Diesel oil	0.86	0.48
Natural gas	1	0.22
Crude oil, kerosene, fuel oil	1	0.23

**Table 4:** The rating grade of model accuracy.

Grade	*p*	*D*
Excellent	≥0.95	≤0.35
Qualified	0.80 ≤ *p* < 0.95	0.35 < *C* ≤ 0.50
Barely qualified	0.70 ≤ *p* < 0.80	0.50 < *C* ≤ 0.65
Unqualified	<0.70	>0.65

**Table 5:** The prediction results of SVR method.

Year	Gasoline (Mt)	Diesel Oil (Mt)	Natural Gas (billion m^3^)	Kerosene, Crude Oil, Fuel Oil (Mt)
2016	2.18	5.37	98.33	0.87
2017	2.36	5.64	108.48	0.91
2018	2.51	6.08	117.25	0.95
2019	2.63	6.48	125.23	0.97
2020	2.721	6.82	131.72	0.99

**Table 6:** The CO_2_ emissions from each energy at the predicted consumption level in 2020.

Year	Gasoline (Mt)	Diesel Oil (Mt)	Natural Gas (billion m^3^)	Kerosene, Crude Oil, Fuel Oil (Mt)	Sum (Mt)
2020	7.95	21.13	22.21	2.98	54.27

**Table 7:** The CO_2_ emissions from coal and coke of Chongqing in 2020 under the energy intensity of Chongqing in 2015.

Year	Energy Intensity	Coal and Coke Consumption (Mt)	Coal and Coke CO_2_ Emissions (Mt)
2015	0.44	60.47	122.64
2020	0.44	107.92	205.08

**Table 8:** The target CO_2_ emissions of Chongqing under the given GDP and emission target.

Year	CO_2_ Emission Intensity	Total CO_2_ Emissions (Mt)	The CO_2_ Emissions from Coal and Coke (Mt)
2015	1.025	161.05	122.64
2020	0.823	205.76	151.48

**Table 9:** The coal consumption and CO_2_ emissions from coal and coke of Chongqing in 2020 under different energy intensity scenarios.

The Average Annual Decline in Energy Intensity (%)	Energy Intensity in 2020	Energy Consumption in 2020 (Mtce ^1^)	Coal Consumption in 2020 (Mt)	The CO_2_ Emissions from Coal and Coke (Mt)
1%	0.42	104.61	100.37	190.74
2%	0.40	99.43	93.12	176.96
3%	0.38	94.46	86.17	163.74
4%	0.36	89.69	79.49	151.05
5%	0.32	85.12	73.08	138.88

^1^ The unit of Mtce means ‘million tons of standard coal equivalent’.
